# Internet of medical things and blockchain-enabled patient-centric agent through SDN for remote patient monitoring in 5G network

**DOI:** 10.1038/s41598-024-55662-w

**Published:** 2024-03-04

**Authors:** Anichur Rahman, Md. Anwar Hussen Wadud, Md. Jahidul Islam, Dipanjali Kundu, T. M. Amir-Ul-Haque Bhuiyan, Ghulam Muhammad, Zulfiqar Ali

**Affiliations:** 1 Department of Computer Science and Engineering, Mawlana Bhashani Science and Technology University, Tangail, Bangladesh; 2 Department of Computer Science and Engineering, Constituent Institute of Dhaka University, National Institute of Textile Engineering and Research (NITER), Savar, Dhaka, 1350 Bangladesh; 3 Institute of Information and Communication Technology, Bangladesh University of Engineering and Technology (BUET), Dhaka, Bangladesh; 4 Department of Computer Science and Engineering, Green University, Dhaka, Bangladesh; 5 Department of Computer Engineering, College of Computer and Information Sciences, King Saud University, Riyadh, Saudi Arabia; 6 School of Computer Science and Electronic Engineering, University of Essex, Colchester, UK

**Keywords:** Internet of things, Internet of medical things, Blockchain, Software defined networking, Patient monitoring, Remote patient monitoring, Healthcare management, Patient centric agent, Mathematics and computing, Engineering

## Abstract

During the COVID-19 pandemic, there has been a significant increase in the use of internet resources for accessing medical care, resulting in the development and advancement of the Internet of Medical Things (IoMT). This technology utilizes a range of medical equipment and testing software to broadcast patient results over the internet, hence enabling the provision of remote healthcare services. Nevertheless, the preservation of privacy and security in the realm of online communication continues to provide a significant and pressing obstacle. Blockchain technology has shown the potential to mitigate security apprehensions across several sectors, such as the healthcare industry. Recent advancements in research have included intelligent agents in patient monitoring systems by integrating blockchain technology. However, the conventional network configuration of the agent and blockchain introduces a level of complexity. In order to address this disparity, we present a proposed architectural framework that combines software defined networking (SDN) with Blockchain technology. This framework is specially tailored for the purpose of facilitating remote patient monitoring systems within the context of a 5G environment. The architectural design contains a patient-centric agent (PCA) inside the SDN control plane for the purpose of managing user data on behalf of the patients. The appropriate handling of patient data is ensured by the PCA via the provision of essential instructions to the forwarding devices. The suggested model is assessed using hyperledger fabric on docker-engine, and its performance is compared to that of current models in fifth generation (5G) networks. The performance of our suggested model surpasses current methodologies, as shown by our extensive study including factors such as throughput, dependability, communication overhead, and packet error rate.

## Introduction

At present, the Internet of Things (IoT) provides valuable support in ensuring proper online-based medical services and its successful implementation has been made possible through the utilization of various IoT device applications. Application of IoT includes wearables and sensors, wireless sensors, and similar devices used to store limitless observation of physiological signs or medical data of patients to servers like cloud-based servers in a secure manner^1^. Compared to traditional medical services, the secure collection and constant monitoring of patient’s physiological signs or medical data through the Internet of Medical Things (IoMT) provide the best possible medical service. This is particularly advantageous for developing countries with a limited number of healthcare service providers or healthcare professionals. The interconnection among medical devices, and software that can communicate via the Internet globally, is essentially referred to as the IoMT^2^. It is the baseline for the remote patient monitoring system. It became popular in the cases of contagious diseases since we need to monitor and make decisions remotely. The collection of physical health-related data can be accumulated by IoMT during transmissible sicknesses like COVID-19^3^. Within this technology, the point-to-point transmission could be anonymous.

However, due to privacy concerns, patients are currently not permitted to communicate their personal health information with hospital administrators through electronic means, thereby hindering a transparent and effective healthcare system. As complex patients seek care from multiple healthcare organizations and providers who utilize different electronic health records, the lack of connectivity becomes increasingly challenging. One potential solution for data collaboration is the adoption of a blockchain-based system, which offers numerous advantages. Nevertheless, blockchain technology is still in its early stages, and significant organizational, legal, and technological constraints impede its full potential in healthcare^4^.

Although the 5G networking system and the Blockchain are two unique technical approaches, there are use scenarios and possible affinities where adding the Blockchain to the 5G networking system might improve networking mechanism. Blockchain authentication provides an immutable record of device authentication, making it difficult for hackers to breach; the key benefit of this integration is offering security and authentication processes within the 5G network system. Again, network segmentation is possible in 5G networks, and in these virtual network slices, the Blockchain may be utilized to govern service-level agreements and guarantee fair compensation for the networking system. Additionally, the Blockchain technique guarantees the decentralized 5G network, where the 5G may run without human involvement to assure network connectivity in the locations where human entry is challenging. In addition, the Blockchain approach can simplify the billing procedure in the 5G ecosystem’s entry points to lower the incidence of billing fraud. By guaranteeing security, billing transparency, data management in the network, and creating more effective user-centric 5G ecosystems, the Blockchain technique may improve the 5G networking scheme. This raises challenging issues in designing an effective, efficient, and secure system. Remote Patient Monitoring (RPM) systems, on the other hand, ensure patient privacy while preserving a significant amount of patient medical data. Privacy entails an individual’s complete control over data streams and the ability to define access level permissions for others. In 2013, a substantial number of well-known medical companies experienced data breaches, reaching a rate of approximately 44%. These breaches continued to escalate, with a reported increase of 60% from 2013 to 2014, resulting in economic losses that grew significantly by 282%. RPM communication is particularly susceptible to hackers due to vulnerability threats and relatively weaker cryptographic techniques compared to wired communication. Threats can originate from insiders, such as healthcare professionals and individuals related to healthcare, as well as external factors like intruders, hackers, and work environments, making the security of healthcare data a critical concern^5^. Most IoT systems use a single centralized server to maintain patient medical data, which renders the architecture vulnerable to a single point of failure when providing healthcare services with a massive volume of end-to-end transmissions Traditional eHealth systems face challenges in delivering proper healthcare due to various types of threats, including Denial of Service (DoS) and ransomware. Some IoT architectures utilize Edge devices, such as smartphone apps, to transmit and store medical data on third-party cloud servers. As these systems rely on wireless technologies such as WiFi, Zigbee, or Bluetooth to maintain data streams, they become susceptible to eavesdropping cyber-attacks, such as DoS, man-in-the-middle attacks, and insider attacks^6^. Because of the involvement of third parties, the traditional Edge or Clouds method cannot assure patient medical data accountability and traceability. The management of patient medical data in a cloud-based IoT architecture necessitates a high level of accountability, transparency, and adherence to numerous health data regulations. Despite the establishment of various health data regulations, it remains challenging to ascertain the compliance of Cloud service providers, posing a threat to the reliability of data management in Clouds. The adoption of Mobile Cloud Computing (MCC) based IoT architecture has improved the capabilities of smartphone apps by enabling them to upload patient medical data to cloud servers. In this context, the efficiency of data processing in the cloud server primarily relies on optimizing latency and minimizing connection loss issues^7^.

Table 1 lists the acronyms with definitions.

**Table 1:** Terminologies are listed alphabetically.

Terms	Description	Terms	Description
ACLF	Assistive Care Loop Framework	LBP	Local Binary Pattern
BC	Blockchain	PCA	Patient-Centric Agent
BSN	Body Sensor Network	PDA	Personal Digital Assistants
CC	Cloud Computing	PM	Patient Management
DoS	Denial of Service	QoS	Quality of Services
GAN	Generative Adversarial Network	RAP	Remote Access Points
HCU	Healthcare Control Unit	RPM	Remote Patient Monitoring
HM	Healthcare Management	SC	Smart Contact
HPW	Healthcare Provider’s Wallet	SDN	Software Defined Networking
IoMT	Internet of Medical Things	SDP	Sensor Data Provider
IoT	Internet of Things	SNAP	Sensor Network Assessment for Patients
LPU	Local Processing Unit	*TC*_*B*_	Time Complexity of Blockchain
MEC	Mobile Edge Computing	*TC*_*H*_	Time Complexity of Holochain
ML	Machine Learning	WSN	Wireless Sensors Network
NFV	Network Function Virtualization		

Mobile Edge Computing (MEC) has been developed to address the need for reduced latency and enhanced real-time application performance, particularly in scenarios requiring high bandwidth. MEC does this by facilitating distributed computing, whereby a range of tasks are executed on edge devices. Edge devices refer to a range of hardware components that include local storage systems, broadcasting network controllers, routers, switches, Remote Access Points (RAP), Base Stations (BS), and hotspots. The MEC system enables a distributed environment by executing activities close to the end user. A variety of edge computing systems have been created, making use of open-source software and hardware, such as Software-Defined Networking (SDN) and Network Function Virtualization (NFV), to improve computing capacity and facilitate virtualization approaches^8^. The current RPM system is vulnerable to various cyber threats or attacks. Therefore, there is a need for an e-health system that can effectively manage, process, and analyze patient-generated medical data while ensuring its accuracy and accessibility to authorized users. Additionally, the medical data should be presented in a manner that is easily understandable to healthcare professionals or physicians. Furthermore, it is crucial for such an e-healthcare system to meet patients’ Quality of Service (QoS) requirements. Presently, researchers are implementing Blockchain^9^ to address these challenges. Blockchain technology offers valuable support in maintaining users’ privacy by providing secure storage access control and facilitating the sharing of medical data without relying on third parties. Hyperledger Fabric is a blockchain framework specifically designed for private, permissioned blockchains. This framework enables companies to restrict access to their information only to those who have been granted permission to view it. It caters to organizations that prioritize privacy and control over their data. The robust permissioned mechanism provided by Hyperledger Fabric allows for flexible assignment of reading and writing rights to different entities within the blockchain network. Participants in the blockchain network are equipped with public and private keys, enabling them to interact with the ledger exclusively on their behalf. Hyperledger Fabric also addresses a crucial privacy concern that other blockchain solutions tend to overlook. Additionally, Blockchain-based e-healthcare architectures have been developed to autonomously manage healthcare medical data, handle user identities and access schemes, facilitate secure sharing and management of medical documentation, and efficiently control patient key management.

When implementing the Blockchain algorithm, such as mining, on resource-constrained devices like wireless sensors and other IoT devices, it becomes even more challenging at the implementation level. The consensus mechanism, a crucial component of Blockchain (BC), refers to the overall agreement among Blockchain Miners regarding the state of blocks. However, incorporating this consensus mechanism into various Blockchain-based e-health systems requires significant computational resources. Gope and Hwang et al.^10^ proposed BSN-Care, a contemporary healthcare system, for Remote Patient Monitoring (RPM). The BSN-Care architecture incorporates traditional devices such as BSN (Body Sensor Network), Local Processing Unit (LPU), and BSN-Care Server. The BSN-Care Server utilizes a heuristic technique to interpret patient physiological data transmitted via the LPU in the BSN-Care system. If the physiological data surpasses predetermined criteria, the BSN-Care Server sends an alert to healthcare providers. Dwivedi et al.^11^ aimed to minimize the cost function on IoT devices by providing an entry point for collecting data blocks from a group of IoT systems. Tuli et al.^12^ introduced a generic broker in a Blockchain-based architecture to distribute tasks among different fog devices, addressing the challenges of computational overhead. However, these approaches rely on centralized Blockchain-based architectures, leaving them vulnerable to cyber-attacks such as tampering and Denial-of-Service (DoS). This paper proposes a patient monitoring system combining SDN and Blockchain to control and secure healthcare data that saves and manages patients’ medical data. Here, medical data include both public and personal medical records, and this process of saving medical records is performed at the time of monitoring a remote patient on an IoT network. The contribution of this paper is mentioned as follows.

- First of all, we propose an IoMT architecture for a remote patient health monitoring system through the patient-centric agent in a 5G network.
- Moreover, we employ three emerging technologies, SDN, Blockchain, and 5G, to control different sensor networks and secure healthcare data in the IoMT.
- In addition, we also utilize a combined SDN and Blockchain technology in the 5G network to preserve the patient’s personal data.

The rest of the paper has been organized as follows: Section “Literature review” describes the literature studies based on eHealth, SDN, and Blockchain. The description of the dataset is described in Section “Dataset description”. We then proposed a remote patient health monitoring system in Section “Proposed system model”. In Section “Implementation and performance analysis”, the performance analysis has been explained adequately. Moreover, limitations and open challenges are covered in Section “Limitations, open challenges and future directions”. Lastly, we conclude the paper in Section “Limitations, open challenges and future directions” with future directions.

## Literature review

This section discusses literature studies based on healthcare architectures that have been implemented conventionally and intelligently in various fields.

### Conventional healthcare framework

The earliest healthcare framework like Codeblue^13^ used a Body Sensor Network (BSN). The proposed system uses medical wireless sensor devices for communicating between end-users and healthcare professionals Medical wireless sensor devices include personal computers, Personal Digital Assistants (PDA), and laptops. Using this healthcare data, some analysis is performed, and based on this analysis, healthcare professionals give a result in an express-inscribe way. Here, the authors discuss the necessity of the safety and secrecy of patients’ medical records. The author also mentioned the secrecy and safety conservancy issues. MEDiSN is a hospital patient monitoring system based on several physiological motes powered by a battery. This architecture highlights some issues of reliable or trusted communication, routing, and QoS. In MEDiSn architecture design, authors mention the requirement of encrypting medical data; however, their research needs to show the encryption process, which ensures reliability and integrity. To monitor the patient’s health condition, Alarm-Net^14^ introduced a heterogeneous network architecture that was developed through BSN and environmental sensors. The proposed system ensures power management and a confidential integrity policy through the use of the circadian activity rhythm module. Also, the authors proposed that Alarm-Net failed to ensure security against leakage of resident’s locations. In another study, MobiCare, a large area-based mobile patient monitoring system designed by Chakravarty, was implemented to collect physiological data situation of patients. This system provides security and privacy for real-time applications; however, it should implement the issue of secure localization.

Sensor Network Assessment for Patients (SNAP) was developed in a similar project to address security threats in cellular healthcare applications. Still, it does not use the verification procedure while providing medical data. Also, according to their architecture, adversaries can easily change or modify original medical data because they lack the use of encryption processes when transferring data in text format to the controller. They also implemented attribute-based authentication mechanisms in healthcare system architectures. Attributed-based authentication denotes the proof of entities such as people in some requirement criteria where a person must have a certain number of attributes, such as a doctor with ten years of experience in heart specialization should be able to access the health data of heart disease patients. A validator assures that a person owns the required characteristics during the attributed-based authentication and encryption procedure. For security and privacy-preserving health information, two attribute-oriented authentication and transmission schemes share in HSNs, which is a social platform where both patients, as well as healthcare service providers, present data records of the medical system, their vision; also, access management can be characterized by a predefined set of characteristics such as user specification, past medical records, and their current social position. Researchers further proposed some methods, like in^15^ authors proposed a method which may allow physicians to make fast decisions in cardiovascular cases for disease prediction.

### Blockchain in IoMTs

**Figure 1:**
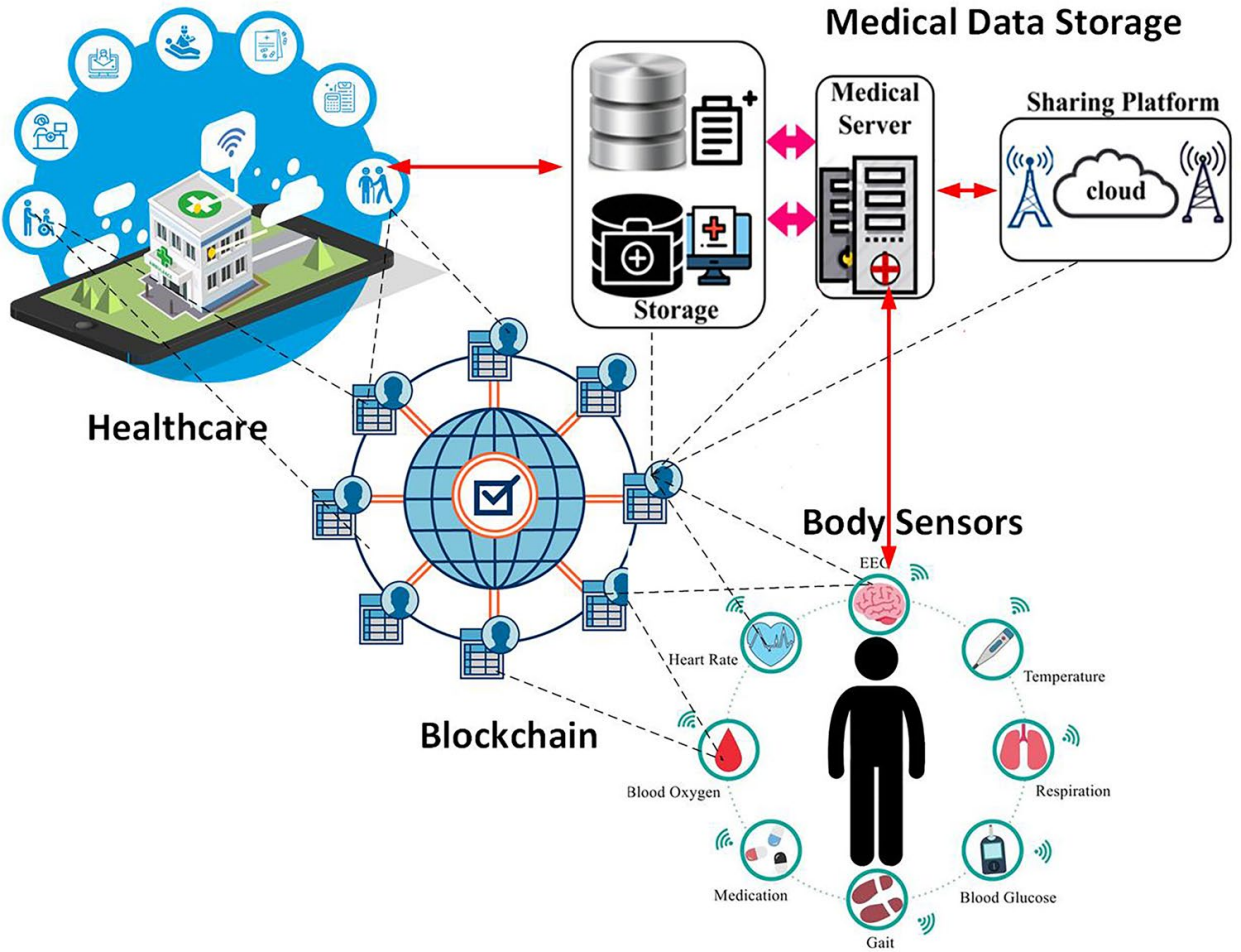
Blockchain in internet of medical things.

BC technology provides tremendous improvement for the solution of different healthcare monitoring architectures because it integrates with AI Kuo et al. proposed a healthcare architecture that uses AI and Blockchain^28^. Using this system, many institutions can share their medical records without compromising security breaches and improve the AI model through training in different medical data. As a result, it enhances performance for better prediction of diseases for various scenarios of the patients. Wang et al. introduced a parallel healthcare architecture that also used AI, IoT, and Blockchain^29^. They proposed this architecture for monitoring a patient’s condition in an instantaneous situation. In this proposed system doctors, patients, and healthcare management professionals can communicate easily through Blockchain, and IoT frameworks and obtain better service with less effort. Figure 1 is the graphical representation of the BC services in the domain of IoMT.

Again, Gaetani et al.^30^ introduce an architecture based on Blockchain that consists of two layers in the Cloud Computing environment. The first layer of the BC stores the record of operations performed by the distributed database. The second layer stores the records of the logged operations created by the first layer database using Proof of Work. Novo et al. proposed a Blockchain-based decentralized architecture that maintains access control for memory and power-constrained IoT devices. Uddin et al. proposed a BC-leveraged decentralized IoT framework for an e-Health care system that consists of three layers. The first layer is called the sensing layer, which transmits medical sensor device data to a smartphone. The second layer, also known as the processing layer (NEAR), is made up of devices that are only a single hop away from data-sensing IoT devices. The third layer called the FAR processing layer consists of the Cloud or other high-computing servers. Furthermore, details on patient agents’ adoption of 5G Architecture through managing the resources of the 5G network to embosom the Blockchain technologies to process health data are discussed.

### Holochain as a distributed ledger for secure and efficient IoMT

**Figure 2:**
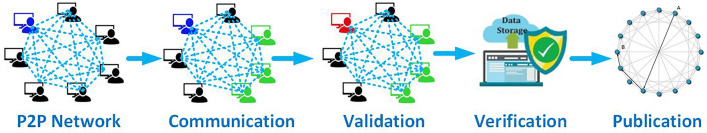
Transaction process of holochain technology.

To automate the preservation of privacy for the IoT services, Holochain is being used in the cloud Holochain is a later version of Blockchain that works with the data more, with data ownership^32^. This is an open-source platform. Peer-to-peer networking protocol is maintained by this open-source software development platform. The transaction process of Holochain is depicted in Fig. 2. Again, Holochain allows the creation of server-less applications where the user can run the application individually on their device, and the storage of data is also at the user’s end but through this framework, they can be connected to each other directly. To make IoMT more efficient, Holochain can be more efficient because it does not require a global consensus procedure. Unlike blockchain technology, users are responsible for confirming transactions by preventing the transmission of data to all nodes. Only certain hosts with the same application running are dispatched in this procedure, and they are responsible for validating the transaction. It features a hashed signature for detecting communication verification to prevent data manipulation. However, from the time complexity analysis of both blockchain and holochain, we get the following equations^33^.

1 $$TC_{B} = BigO(nodes^{2} \times t)$$

Here in Eq. (1) the time complexity of the blockchain is calculated. Here, t is the transaction required.

2 $$BigO(t \times (log(nodes) + complexity))$$

The runtime complexity of holochain is represented in Eq. (2), where t is the number of transactions required and the complexity varies by application. Thus, it performs different operations in less time compared to the blockchain but the currencies are optional in Holochain. Panwar et al. studied various types of distributed ledger technologies and discussed how blockchain has evolved over time^34^. They proved that blockchain is not the only distributed ledger technology. When it comes to the security of Artificial IoMT networks, Holochain could be a choice of researchers since the technology has been utilized in the cloud.

### State-of-the-art SDN-blockchain enabled ehealth systems for patient monitoring

Nowadays, leading technologies like SDN and Blockchain have tremendous contributions to the e-healthcare system SDN helps to control the sensitive data plane-wise, and BC offers security and confidentiality for the desired system efficiently. The authors revealed the merits of each technology and its performance within the framework. A monitoring system for healthcare was proposed with the trust management system through SDN and Blockchain by Barka et al. Blockchain was considered as the back-end monitoring^35^. Lin et al. proposed an architecture to maintain big data for emotion-aware applications developed through SDN and 5G technology. Here, the authors discuss details about the working process of transmission, storage, and data collection^36^. According to this design, data is transported across the data plane of the SDN with high throughput and then stored in the data center by the SDN application. Data is also uploaded to the cloud at the end of this execution process^37^. In another IoT-enabled study^38^, Sahoo et al. offered a three-factor IoT network authentication system for healthcare system applications with secure information channels. They proposed the TFASH and ECC scheme, which is capable of providing a high level of security. The research should further consider more parameters for attack minimization.

In summary, from the above discussion, researchers presented different techniques to solve the problems regarding the smart healthcare monitoring system. Table 2 presents the existing technology and its associated research gaps. Also, they proposed some smart contract systems to contract patients and doctors very confidentially. Motivated by the above research work, this paper focuses on the remote patient healthcare monitoring model through the emerging technologies SDN and Blockchain. Furthermore, we apply the PCA technique for smart contracts efficiently. Additionally, we have considered Table 3 to discuss the major issues and challenges of the existing work.

**Table 2:** Emerging technologies and research gap.

Works	Technologies Used					Research Gaps
	Cloud	e-Health	IoT	SDN	BC	
Deebak et al.^16^	✓	✓	✓	✕	✕	The energy consumption estimation and the time requirement have not been analyzed in this article
Koteluk et al.^17^	✓	✓	✓	✕	✕	The model is explained, however, it should be discussed in a real-world scenario
Yang et al.^18^	✓	✕	✓	✕	✕	Data security should be addressed properly in this work
Li et al.^19^	✓	✓	✓	✕	✕	Time management and data heterogeneity are not properly addressed
Gerli et al.^20^	✕	✓	✕	✕	✓	Attacks that can happen in the e-healthcare system have not been taken into account in this research
Zou et al.^21^	✕	✓	✕	✓	✓	It lacks an analysis of implementation cost
Consuegra et. Al^22^	✕	✓	✕	✕	✕	Security and privacy are not addressed in this work
Ccalhan et al.^23^	✕	✓	✓	✕	✕	The possibility of attacks is not considered in this work
Rahman et al.^24^	✓	✕	✓	✓	✓	The decisive analysis should have been discussed based on the distribution process. Also, in the result analysis part, more parameters are needed to be considered
Mahmoud et al.^25^	✕	✓	✓	✕	✕	Transparency of data, the possibility of attacks in the network, and privacy measures are not considered in this work
Ahmed et al.^26^	✓	✓	✓	✕	✓	It lacks controlling and monitoring IoT sensors. Also, Time management and data heterogeneity were not properly addressed
Sireejaa et al.^27^	✕	✓	✓	✕	✓	It lacks controlling and monitoring IoT sensors
Our proposed system	✓	✓	✓	✓	✓	–

**Table 3:** Existing works: issues & challenges.

Articles	Issues & challenges
Kaur et al.^39^	Ensuring Data Privacy Congestion in the network
Hu et al.^40^	Latency Minimization Fast delivery of critical Data Data Security
Ghaffar et al.^44^	Unpredictability in the network Enormous information flow Diverse communication
Jaiswal et al.^45^	Data Confidentiality Data pass without Alteration Authenticity Refusal from the sender side Tolerance for errors Limited Memory & Energy
Vahdati et al.^46^	Unauthorized Access Complicated data transfer
Magsi et al.^47^	Power Failure and Consumption Network Failure
Awotunde et al.^48^	Data Protection Sensor Failure Data Availability Diversity of Sensors
Juyal et al.^49^	Location Privacy Resource Management Eavesdropping attack
Kushniruk et al.^50^	Data security Trust management
Lee et al.^51^	Storage Management damage management in case of any failure Time and Space management

## Dataset description

The data collecting capability utilizes Internet of Things-enabled sensing devices to gather real-time tracking medical data from patients. Sensor technology advances in terms of data handling in biooptic sensors including EEG biotelemetry, electrocardiogram (ECG) sensor, blood pressure checking, heart rate monitoring, insulin monitoring, virus monitoring, and healthcare surveillance. It demonstrates how ubiquitous, on-demand access to a common pool of customizable computer environments may be implemented. Table 4 shows the sample dataset where the patient ID represents the detailed information of a patient, the doctor ID represents the doctor’s information, including the doctor’s prescription for a particular patient, and the test code presents the medical examination report on a specific date. Table 5 summarizes the core functions required to acquire data from SDN-based patient-centric agent devices. We also made all the materials with datasets via Hyperledger^1^. For testing purposes, we collected 1000 medical data where 15 doctors “prescribed 100” patients with 20 different test cases. Due to privacy issues, we only collected index numbers from the hospital database.

**Table 4:** Sample dataset with test case description.

Patient ID	Test code (description)	Doctor ID	Date
92	59 (Post-breakfast blood glucose measurement)	6	2020-01-03
84	48 (Unspecified blood glucose measurement)	3	2020-01-07
34	34 (NPH insulin dose)	14	2020-01-25
93	71 (Unspecified special event)	7	2020-01-30
34	68 (Less-than-usual meal ingestion)	11	2020-02-25

**Table 5:** Data collection functions.

Function name	Description
SD_Aceept (device), SD_Drop (device)	Add/remove a sensor to/from IoT HEALTH-CARE
Reset (device), Enable (device), Disable (device)	Delete unused data, enable/disable sensor device
getData (device), put-Data (device)	Take medical data & send data to network
checkSignal (device, nID), skipSignal (device, nID)	Check sending signal to network identifier(nID) and skip signal to send another nID

## Proposed system model

**Figure 3:**
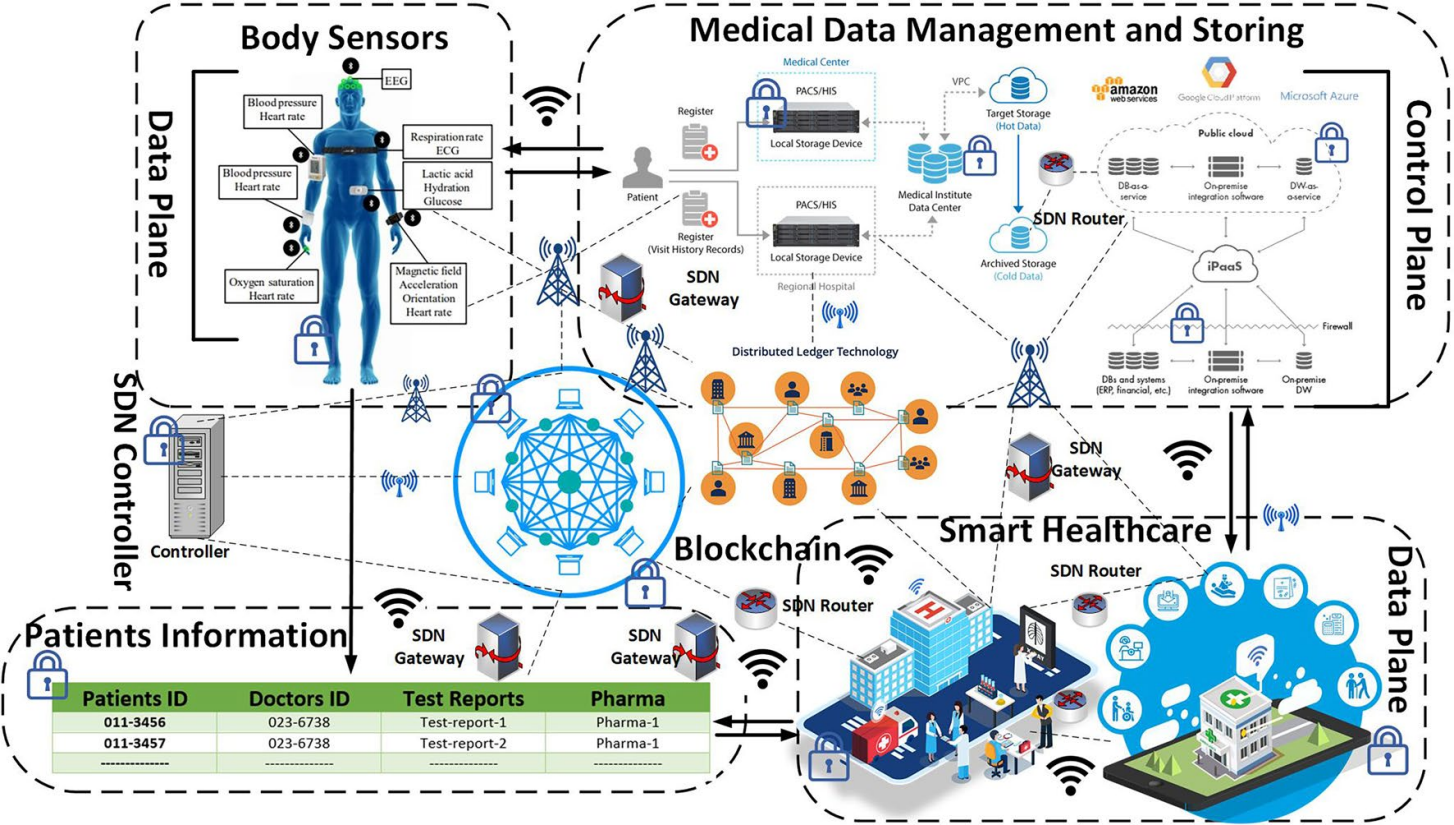
Proposed internet of medical things for patient healthcare monitoring system.

The proposed model of this article is mainly designed based on two emerging technologies, including SDN and Blockchain with different sensor devices, to heighten the security of the IoMT networks, as depicted in Fig. 3. These technologies incorporate a healthcare monitoring system and PCA suitably. Then, the authors divide the full proposed model into several phases, such as sensor data provider with IoMT forwarding devices, combined Blockchain with SDN environment, and the role of intelligent contract through PCA with authentication of BSN technique and private Blockchain approach, logical flow of remote patient monitoring system, and finally, we cover the security analysis phases of the system model. The SDN is a crucial technology that improves the performance of the 5G network in a number of ways, including the creation of many virtual networks inside of a single physical system, which is a key component of 5G networking. SDN makes it possible to slice the network plane by separating the control plane from the data plane. Again, SDN is a networking technology that can help a network modify how it operates in real-time, increasing the speed of 5G networking data transfer and lowering the latency of IoT device connectivity. Additionally, the SDN’s central management mechanism helps optimize traffic flow and lessens congestion in the 5G network because of the massive volume of data traffic present there, which might cause congestion if improperly handled. In addition, SDN is used in the 5G networking system to manage resources and control traffic, as was already mentioned. SDN is therefore used to intelligently regulate the system’s overall energy usage^53^. In summary, SDN plays a significant role in the 5G ecosystem, such as dynamic network management, centralized control, network slicing, flexibility and agility, service orchestration, efficient resource Utilization, and reduced operational costs.

### IoMT forwarding devices with sensor data provider

The IoT devices learn to process the acquired data utilizing IoMT models. Many devices and systems have evolved in the medical domain, including different wearable electronics and other hardware and software. IoT plays a lively function when it comes to real-time remote healthcare systems. A brief introductory description is given here for the communication technologies:

#### IoT forwarding devices

IoT forwarding devices are used to forward patient data through the SDN controller using the PCA^52,53^. The following subsections describe the functionality of various IoT devices such as BSN, SDP (elaborately known as Body Area Sensor and Network Sensor Data Provider, respectively), and so on.

#### Body area sensor network (BSN)

BSN devices are used to create short-range transmission networks. Common BSN devices in healthcare systems are motion trackers, electroencephalography (EEG), electrocardiogram (ECG), and human-computer interaction (HCI) for continuous capture of information. Additional antenna requirements for BSN communication are useless, and power consumption can be reduced by 0*.*1*–*1*.*0 mW.

#### Sensor data provider (SDP)

Various types of software work on a cellphone or mobile devices are considered as sensor data providers (SDP). SDP transmits patient physiological data such as EEG, ECG, BSC, etc., to PCA We presume that each patient uses a mobile device that can receive information about their health condition from various BSNs and sends this information to PCA via SDP.

#### SDN-IoT enabled gateway and patient centric agent

**Figure 4:**
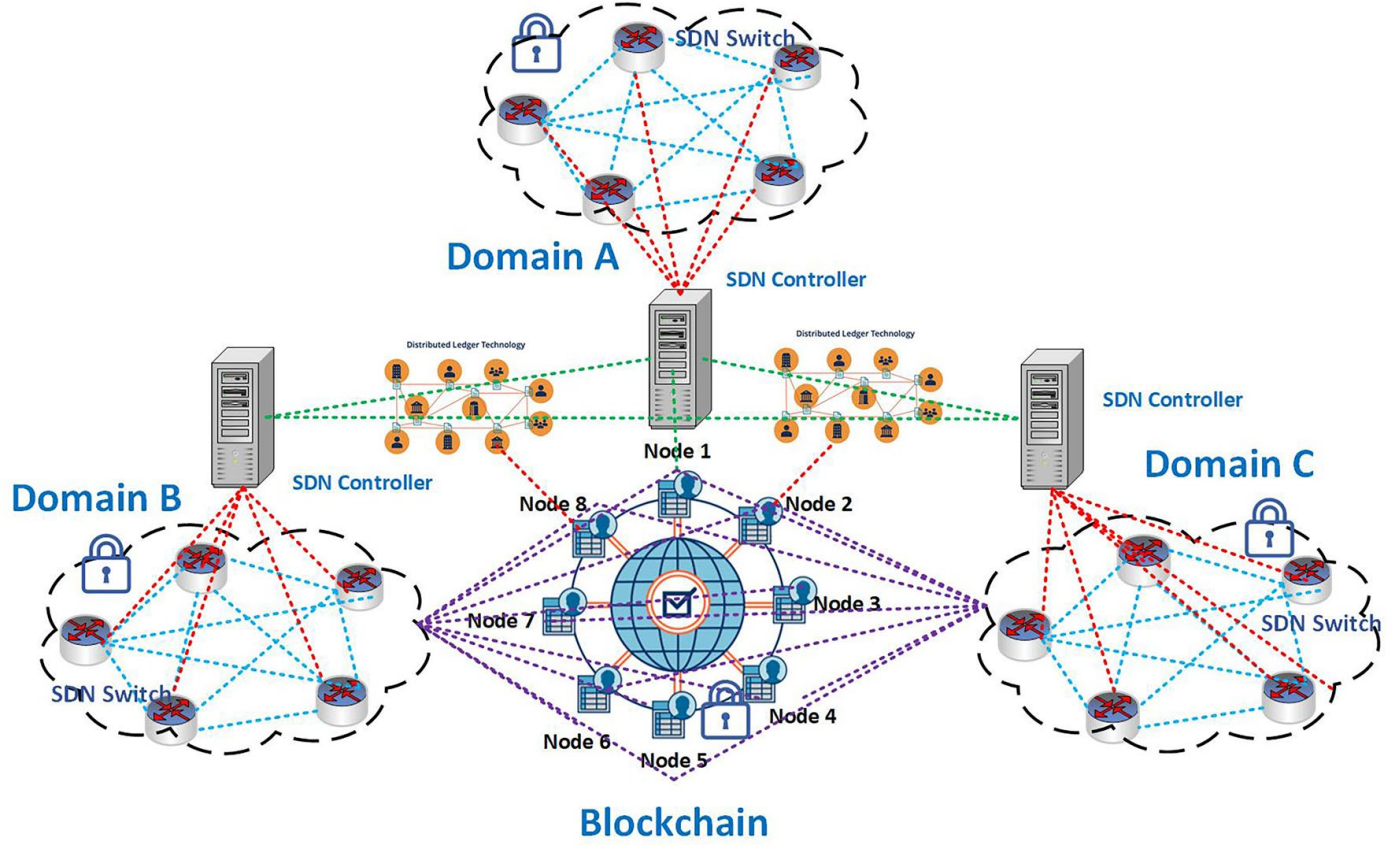
Data transmission scenario in SDN with blockchain system.

In remote patient monitoring, a software-defined network has been used to design healthcare systems where the Blockchain network has been placed between the data plane and the control plane, as depicted in Fig. 4. The domain-wise organization of SDN is adding a novel direction to the desired system. The controller of the SDN is incorporated into the Blockchain approach, which can be capable of providing a high bond of security in the proposed system efficiently. Moreover, the Blockchain network is used not only to store public and private patient data but also to store healthcare information. On the data plane, various IoT devices forward data to the SDN gateway, commonly known as the SDN-IoT gateway^37^, where the SDN controller plane oversees the entire communication and lays down rules for different groups such as Patient-Centered Agent (PCA), Healthcare Control Unit (HCU).

### Strategy of combined blockchain with SDN networking

In this section, we incorporated the SDN network and Blockchain network, which acts as permission or permission-less Blockchain for the patients. However, SDN is prepared by several planes, such as the data plane, control plane, and application plane^54^ as shown in Fig. 4. Moreover, it is a networking paradigm that is capable of offering centralized controllers for any operational data efficiently. These SDN controllers provide scalability, flexibility, and privacy, configure the network and network services, and enhance the network’s performance appropriately. For this model, SDN’s role is to allow efficient support for the streaming of data together with a saved area of this huge data^52^. Furthermore, it includes IoT forwarding devices such as routers, switches, firewalls, BSN, SDP, and Healthcare Provider’s Wallet (HPW).

**Figure 5:**
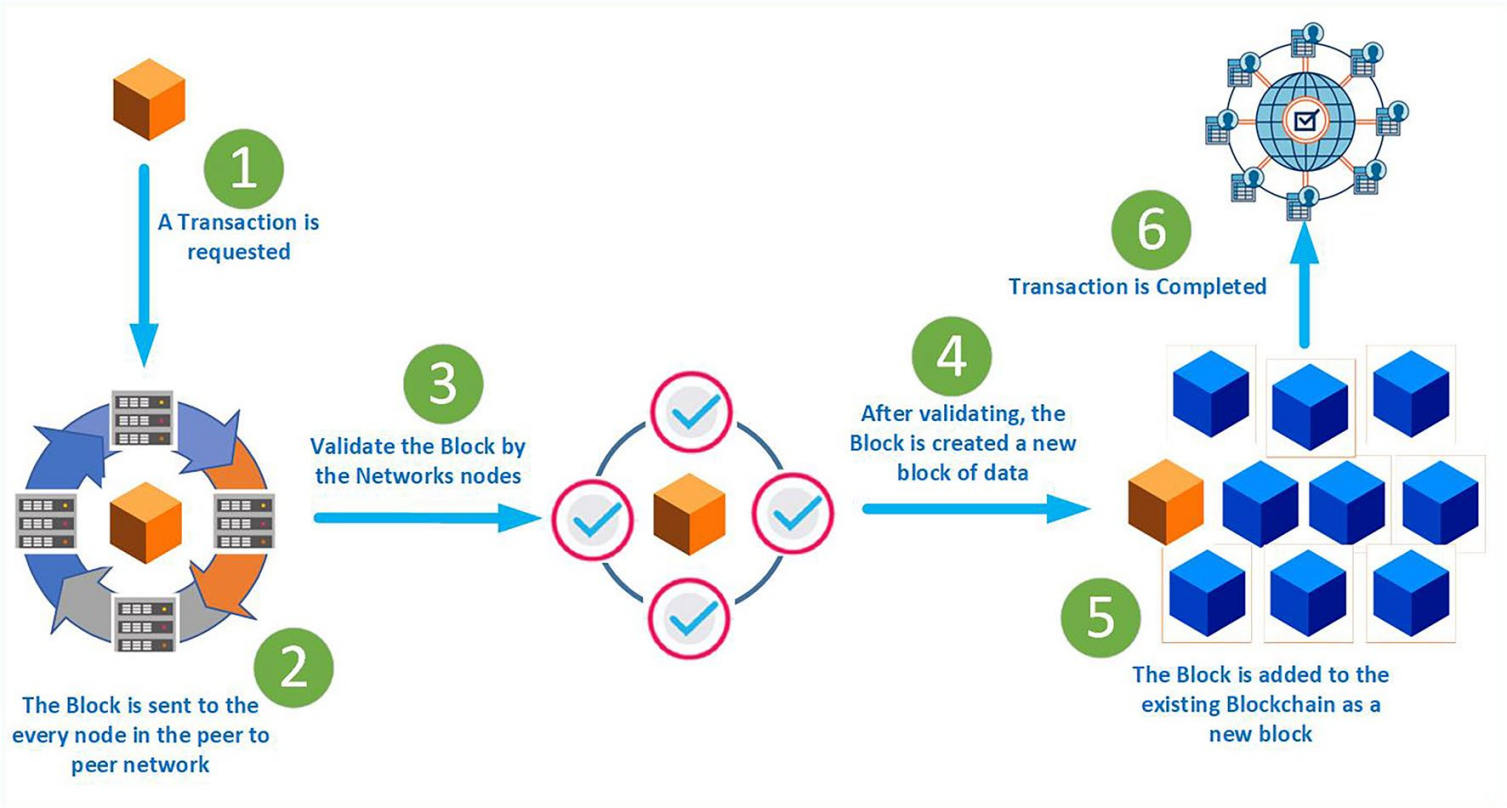
Blockchain transaction process.

Bitcoin’s integrated technology on the other hand, is Blockchain. Because of its nature, it is known as a distributed ledger^55^. Furthermore, it is a distributed and immutable list of elaborating blocks that holds data in the form of a transaction, as depicted in Fig. 5. In the proposed system, this network efficiently manages and controls healthcare professionals or providers. In this architecture, mainly Healthcare Control Unit (HCU) performs the role of the Blockchain. Further, Blockchain networks manage patient data by creating a private chain of the block and monitoring PCA and HPW.

### The role of patient centric agent (PCA)

The main role of PCA is to handle a blockchain component to maintain anonymity when body area sensors send data to the SDN domain Algorithm 1 shows the working methodology of the patient-centric agent, and the following subsections describe the PCA functions in detail. PCA can operate in a computerized device like a laptop, desktop, or server. PCA initiates a session to communicate with the SDP, BCN, and Blockchain Cloud for each task. First, PCA uses public/private keys to decrypt SPD/BCN data and authenticate the trusted source. Then perform various actions based on the results of getAgentNodeModule. PCA architecture has one kind of node called the patient agent node. This node consists of three modules which are SSM, DMM, and MMM^56^. The getAgentNodeModule function sends different action modules based on Patent ID (Pid), Doctor ID (Did), and Patient and Doctor Status as Status ID (Sid). Here, SSM stands for Security Service module, DMM stands for Data Management Module, and MMM stands for Miner Management Module. This agent node has a significant contribution to overall architecture, and one of its modules is called DMM, which is used to classify patients’ medical data. The category classification can be labeled as Normal, Eventful, and Uneventful. The patient’s categorical data, which is uneventful internallysaved in the DMM module. It can also transfer this data to the Cloud when healthcare professionals require it. PCA can also act as a Miner through its MMM module for a specific time if none of the miners performs their execution The SSM module of PCA repeatedly analyzes the vulnerability of the different communication channels. It also checks network security threats when communicating different channels like BSN to SDP, SDP to PCA, and PCA to BSN^10^.

**Figure:**
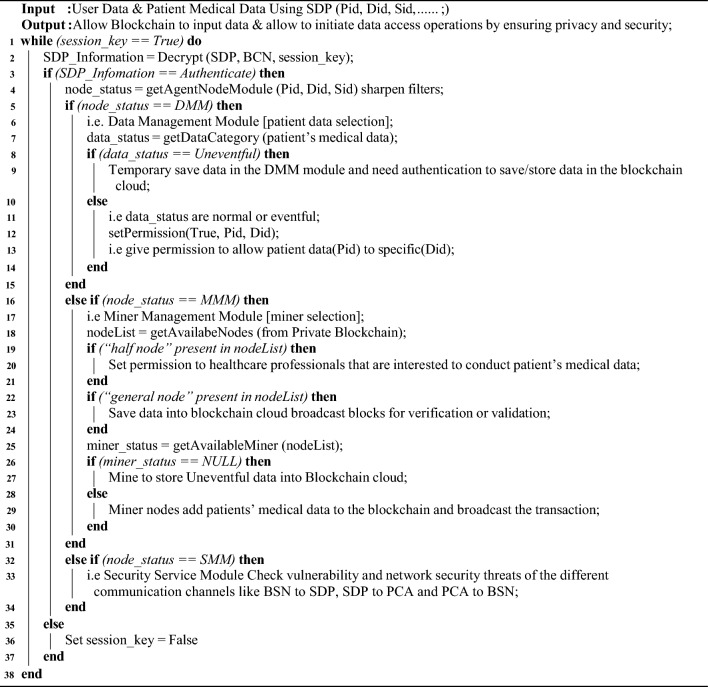
Algorithm 1: Proposed Algorithm of Patient Centric Agent.

#### Authentication BSN to PCA

**Figure 6:**
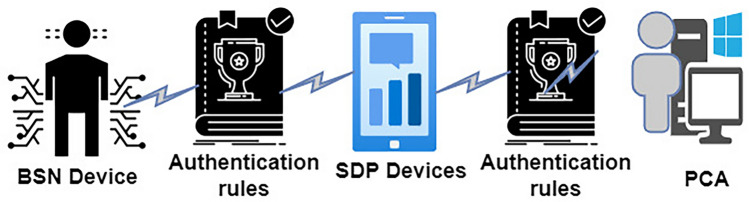
BSN to PCA authentication.

Patients’ wearable medical sensor devices transmit physiological data received by SDP. The confirmation of receiving the patient’s physiological data is done through the lightweight authentication protocol. Here, it generates a key (Ki) known as a sessional symmetric key through BSN and SDP devices. This key ensures authenticity. It is successfully done using a mechanism. This mechanism is known as dynamic key generation. The authentication process of BSN to SDP is shown in Fig. 6. At first, the BSN device begins the authentication process through transmission. It transmits to the SDP device. Then SDP device takes flight through an insecure channel from BSN. It also decrypts the information with a given acknowledgment that the authenticity of HMAC is successful^58^. After that, the SDP device verifies the voice difference between BSN and itself. It does the confirmation of whether the voice of BSN is similar to the saved voice of SDP. For this purpose, PCA applies some public/private key pairs The validation of the new public/private key pair is maintained by PCA and SDP devices. In this case, the session key was utilized to authenticate the new public/private key in the authentication method (Ki). Actually, it is needed to avoid the third-party trusted center because this mechanism is enough to ensure the verification of the public/private key.

#### Patient centric agent to private blockchain

Communication between PCA and BSN devices is performed through a modified Blockchain network. Miner selection and patient data selection, that is, which data should be stored in Blockchain are performed by PCA. Distributed databases like Blockchain can play a significant role in maintaining patients’ medical data with safety and security. It is also considered a trusted platform because all nodes that execute in this Blockchain network are verified All nodes in the Blockchain network should be supplied by healthcare providers, individuals, or other organizations. Nodes are labeled with some categories in this private Blockchain network. The categorical label of the nodes can be expressed as general, benign, half, and miner nodes which is a kind of Bitcoin Blockchain. Here, healthcare professionals, healthcare providers, or individual users are labeled as half nodes and are interested in conducting patient’s medical data that is saved in the Blockchain network. Nodes that are labeled as general nodes have significant functionality. It can save a chain of blocks. It can also broadcast blocks for verification or validation^59^. Some other nodes which are labeled as miner nodes have a more significant role in this architecture. It can perform like a CPU processing operation for block mining. Through the verification process, data packets are made from a legitimate node, and this process is ensured using Miner and general nodes.

### Logical flow execution of proposed remote patient monitoring system

**Figure 7:**
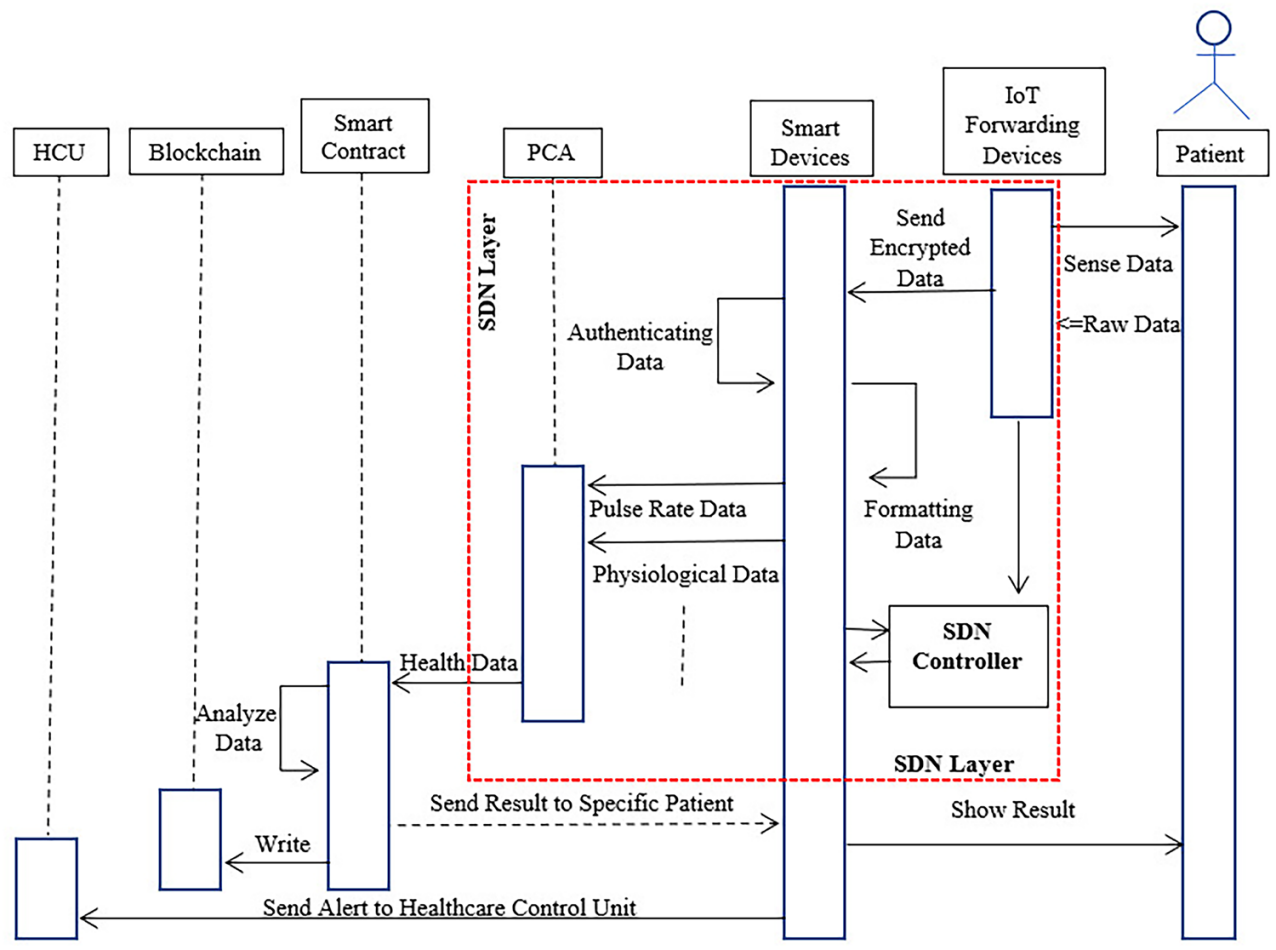
Logical flow execution of the system.

According to the flow model, which is shown in Fig. 7, the user’s data is collected via IoT forwarding devices. The data being collected is raw data that requires processing for further usage. These data are transmitted to the PCA that handles data processing in order to be transferred to a specific contract. The data are encrypted to ensure the security of the information being passed. Each contract is connected to a certain action via the sensing (smart device) device. These are the wearable devices most of the time that process and perform an identification procedure on the data. The authentication layer, which comprises software patterns to convert the data to the sensing device, facilitates this procedure between the user and the primary contract. The PCA layer produces health records such as– data on the pulse rate, physiological data, neural data, and so on, combinedly known as the health record or health data^60^. The analysis of the records is carried out through the smart contract and the analysis of the Blockchain by writing. By adding new data to the Blockchain, a smart contract at the access layer interacts directly with the Blockchain. At the same time, this contract examines the data to see if it contains the proper characteristics for inclusion in the Blockchain. The data is sent back to the smart devices. If an emergency situation arises (as determined by smart contract regulations)^61^, the smart device will send an alarm straight to the Hospital Control Unit (HCU) to guarantee patient safety. The patients, through this way, can see the results that are shown by the smart devices. In this flow model, the Blockchain stores the data in a block so that the data are not being altered. The smart contract performs the task of analysis that is important to identify or control the management system.

#### Risk analysis of the proposed model

There are several risks related to Blockchain technology in the proposed Model. These include:

*Loss of Private Keys*: In case of key loss the user of the system can not retrieve the password as there is no option for key recovery. Thus in this scenario, there is a big chance of losing control of user data^41^.

*Security Threats*: The Blockchain technology is popular for ensuring security in any system, but still, it is not immune from hacking by third parties. Smart contracts and code flaws can be exploited, resulting in security breaches and financial losses. Furthermore, 51% assaults might jeopardize the integrity of some smaller Blockchains^7^.

On the contrary, there are several attacks possible in the 5G network^1^; for example–

*Jamming Attack*: Attackers may deploy jamming equipment to interfere with 5G signals, interrupting the connection to the network as well as causing disruptions in service.

*Attacks by Rogue Base Stations*: Attackers can build up rogue base stations that look exactly like authorized ones. This can result in a device being connected to the rogue station, enabling eavesdropping or other unauthorized operations.

*Man-in-the-Middle (MitM) Attacks*: In 5G, intruders have the ability to intercept and alter data passing between the user and the network, perhaps listening in on conversations or inserting harmful information.

#### Mitigation of blockchain-related risks and weaknesses

To ensure the security of the private keys in this work we have used the SHA-256 (hash method). Here in this method, the two-factor authentication system is integrated. This helps to generate code that has two parts, including the private key and the code generated from the authenticated device. By using robust encryption methods to safeguard data at rest and in transit. The use of public and private key cryptography is essential for safeguarding data and transactions. Again, in order to mitigate security threats regular monitoring of the network system is essential to detect any malicious activities. Further, by ensuring the decentralized method the single-point failure of Blockchain can be mitigated. Through updating the blockchain software on a regular basis to correct known vulnerabilities and enhance the consensus mechanism^62^.

#### Mitigation of 5G-related risks and weaknesses

The use of blockchain to mitigate 5G-related hazards and vulnerabilities is a potential method that leverages the characteristics of both technologies to improve privacy, trustworthiness, and dependability in 5G networks^42,43^. Here are a few ways that cryptocurrencies can assist in addressing the difficulties related to 5G:

*Authenticity and Stability*: For devices with 5G connectivity and clients, blockchain can provide a decentralized and counterfeit-proof identity management solution. This can lessen the danger of identity theft and unauthorized network access. On the other hand, a blockchain’s smart contracts may enforce privacy-preserving communication protocols, guaranteeing that only authenticated devices can access the 5G network and prohibiting eavesdropping and other unlawful activities^42^.

*Confidentiality Safeguards*: Blockchain technology may be used for encryption as well as protection of user data, allowing only authorized parties accessibility. This is essential in 5G networks, which require a large quantity of personal data interchange. Again, Blockchain can handle user permission and data-sharing choices, allowing consumers to have more control over their data and how it is utilized in the 5G network^43^.

*Resilience of Networks*: By motivating healthcare professionals along with patients to contribute their network resources (e.g., bandwidth, computing power), Blockchain can allow the building of decentralized 5G transmission networks. This can boost network resilience, particularly in the event of network disruptions or assaults. Because Blockchain is a distributed system, it is resistant to single points of failure. This can aid in the maintenance of network availability and dependability^2^.

#### Security analysis of the proposed model

We have analyzed three CIA security goals for the proposed model. These are:

*Confidentiality*: The term “confidentiality” describes the safeguarding of private information from unwanted disclosure. In the remote patient monitoring system, confidentiality is crucial to ensuring that only authorized individuals can access sensitive information. Blockchain technology offers a cutting-edge method for achieving remote patient confidentiality. Cryptography is used by Blockchain to protect data on the network. Every transaction on the Blockchain is encrypted, and only participants with private keys have access to the data. This makes sure that sensitive information is kept private and that only those with the right authorization can access it^62^.

*Integrity*: Data authenticity and dependability are referred to as data integrity. This includes the upkeep of patient data over its full life cycle as well as ensuring its validity and reliability. Blockchains’ core building block, the Merkle tree, uses cryptographic hash functions. Merkle Trees offers Blockchains the hash-based design necessary to ensure the integrity of patient data as well as a safe means of data integrity verification^63^.

*Availability*: Data availability analysis is another security goal that refers to the guarantee that all complete nodes have had access to and verified the entire set of patient activities linked to a given block. It does not imply that the data will always be available. The fundamental design of Blockchain architecture prioritizes data availability over data retrievability. A small number of archive nodes managed by outside parties may offer data retrievability, or decentralized file repository systems like the Portal Network can be used to spread data around the network^62^.

## Implementation and performance analysis

### Performance metrics parameters

We have considered four parameters such as reliability, accuracy, throughput, and communication overhead to measure the execution of the preferred performance.

#### Reliability

The level of Reliability of a network refers to its ability to be deemed trustworthy, consistent, and dependable. Moreover, the assessment of a network’s Reliability is based on the incidence of failures and the duration of the recovery process. Overall, measuring the network’s Reliability during catastrophic events is commonly referred to as the network’s robustness.

#### Accuracy

Accuracy is measured to define how close the data is to the actual value. It is calculated by dividing the number of predictions that are correct by the total prediction. We have employed Eq. (3) to calculate accuracy.

3 $$Accuracy = \frac{CP}{P}$$

where CP correct prediction, P total prediction.

#### Throughput

The throughput of a data transmission network paradigm is the amount of data that is successfully moved from one location to another over a given period of time. We have evaluated the throughput by the equation Eq. (4).

4 $$Throughput = \frac{{\sum\limits_{n = 1}^{N} {CBR_{rece} } }}{Simulation \, Time}$$

where CBR Constant Bit Rate received, and *N* is the total number of execution.

#### Communication overhead

Communication overhead guides the supplemental data required for data transmission, including data framing, timing information, and error detection & correction. Firstly, data framing is a crucial process that guarantees data’s accurate transmission and reception. Secondly, in order to guarantee the proper transmission of data and synchronization between devices, it is imperative to include timing information. Finally, implementing error detection and correction mechanisms, such as checksums and re-transmissions, may significantly increase network traffic overhead.

### Environment setup

We implement the proposed architecture/model on Ubuntu 18.04 OS with Intel^®^ Core TM i5-6500CPU @3.20GHZ machine, including 8 GB RAM. Also included Docker Compose, Docker-Engine, Visual Studio Text Editor, NPM as well as Node.js. To implement three-tier networks as peer and server communication, here apply three Docker Containers^64^. The aforementioned system configuration shows that for a single transaction, it requires about 0.1 s to finalize and broadcast a block within the overall network. Here, the observation result shows that the execution time of the proposed system is comparatively lower than the Ethereum network. It seems the proposed system is comparatively faster than the Ethereum network. Table 6 presents an overview of our simulation environment.

**Table 6:** Simulation environment.

Attributes	Parameter names	Values
General parameters	Packet analyzer	Wireshark
	Emulator	Mininet-WiFi
	Cloud storage platform	OpenStack
SDN parameters	No. of SDN controllers	2
	Open flow switches	4
	Gateways	2
	SDN routing protocol	OpenFlow
Blockchain parameters	No. of transactions	Variable
	Block header	80 bytes
Other parameters	Simulation area	1000 m × 1000 m
	No. of IoT devices	1–30
	Simulation time	600 s
	Data rate	10 Mbps
	Initial energy values of IoT devices	12–15 j
	Initial trust values	5 j
	Node transmit packet size	100–512 bytes

### Performance measurement

**Figure 8:**
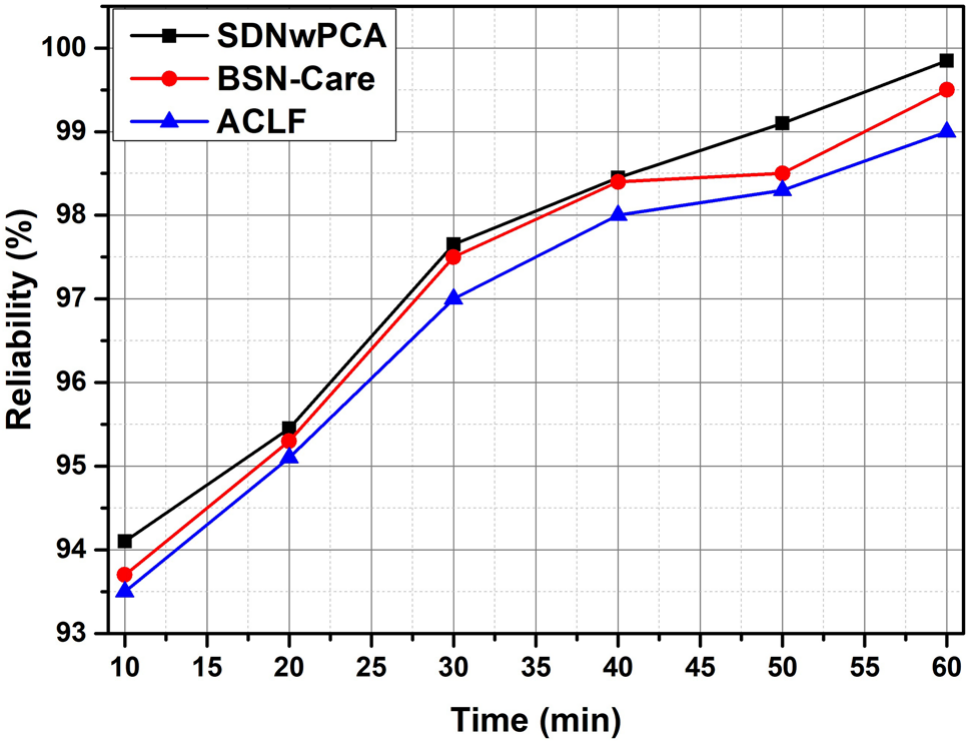
The comparison of Reliability of the proposed model vs. BSN-Care and ACLF baseline with respect to processing time (min) slots.

We display the comparative study of performance analysis of our protocol (named SDNwPCA) with Assistive Care Loop Framework (ACLF) and BSN-Care in terms of reliability, several error packets, and throughput. From Fig. 8, because of our lightweight proximity-based authentication and multilayer storage, our assessment of the proposed safety protocol’s dependability increases over ACLF and BSN-Care (Patient Local Server, Cloud, and Blockchain).

**Figure 9:**
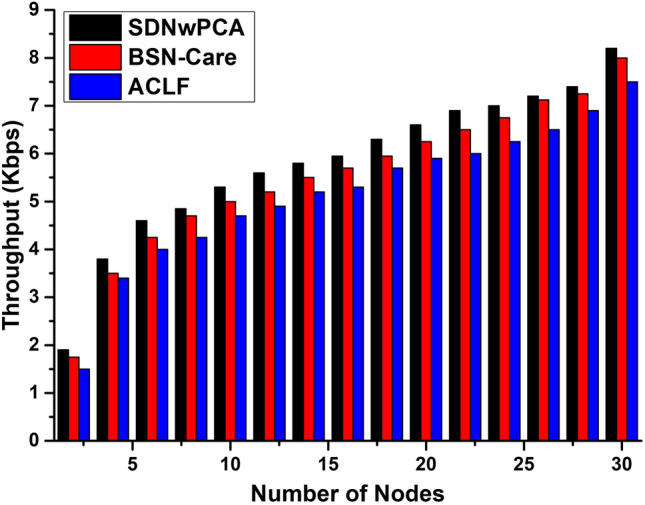
The overall Throughput of the proposed architecture vs. BSN-Care and ACLF baseline with respect to the number of nodes (30).

**Figure 10:**
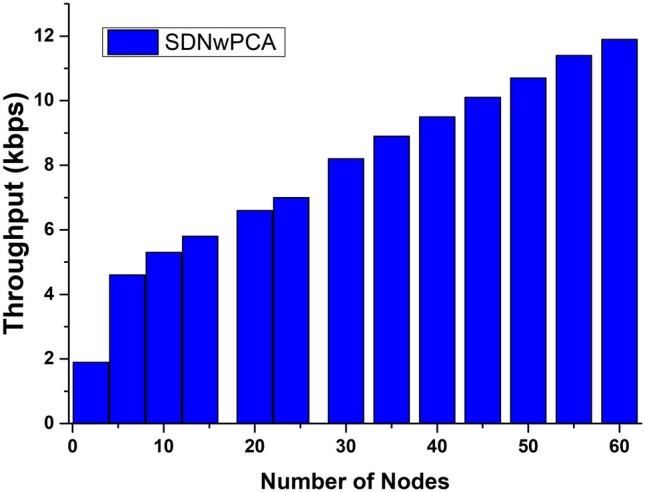
The overall Throughput of the proposed architecture with respect to the number of nodes (60).

The architecture of the ACLF used a triple key feature for making lifelong activity of the sensor. Hence, if the keys are breached, then any intruder can easily get overall control of the devices. To avoid this issue, ACLF slows down its data transmission rate. That’s why overall throughput is lower, and this issue is depicted in Fig. 9. In BSN-Care architecture, they implement their model using a single server. This server is used to save all kinds of records. That’s why there are network congestion issues during transmission. It also decreases throughput. Both ACLF and BSN-Care employed only 30 nodes for their data transmission rate measurements. Consequently, our proposed SDNwPCA model also employs 30 nodes to compare its performance with existing models, as illustrated in Fig. 9. The proposed SDNwPCA model utilizes the 5G network, which enhances high-speed connectivity and increases throughput when compared to all existing models. Fig. 10 presents the comprehensive throughput data for 60 nodes, revealing a consistent increase in data transmission rates as the number of nodes rises. This growth is attributed to the presence of more miners, which significantly alleviates network congestion.

**Figure 11:**
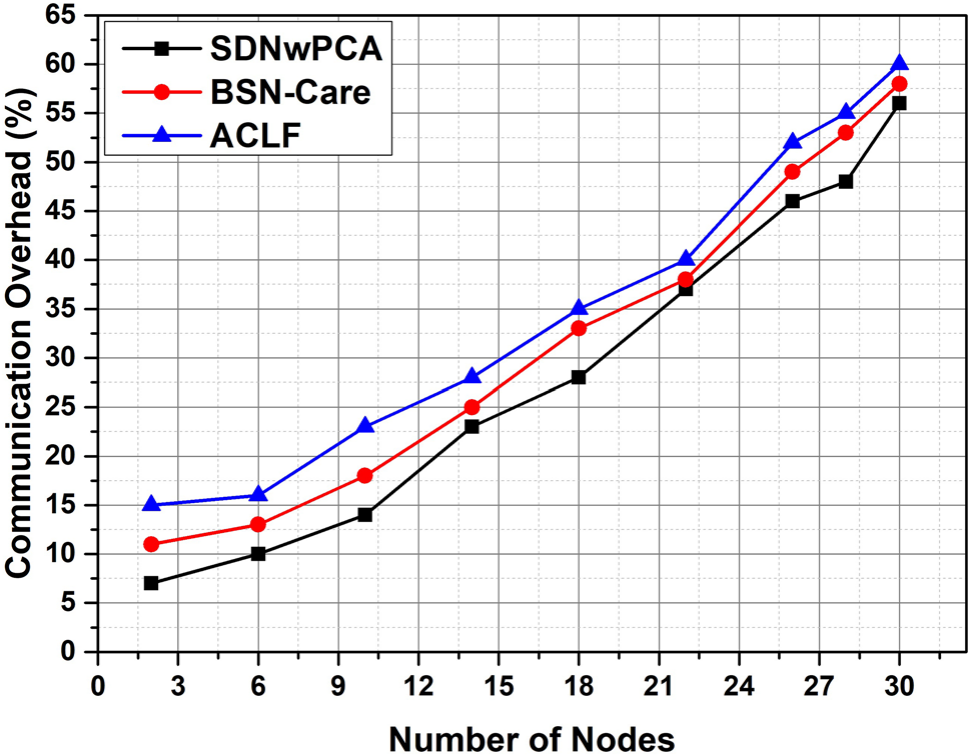
The comparison of communication overhead of the proposed framework vs. BSN-Care and ACLF baseline with respect to the number of nodes.

**Figure 12:**
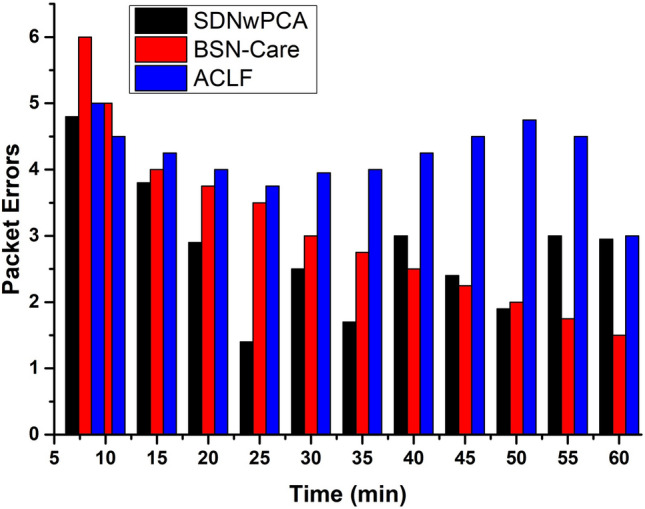
The comparison of Packet Error of the proposed system vs. BSN-Care and ACLF baseline with respect to processing time frames.

In SDNwPCA architecture, we proposed a key mechanism that maintains a continuous-time session. Here, an attempt was made to avoid information sharing, however, this will happen only during deployment. This mechanism ensures the safety of the devices from attackers and reduces overall communication overhead, as shown in Fig. 11. Another issue is locality-based authentication which is not mentioned in the security mechanism of ACLF and BSN-Care architectures. SDNwPCA architecture used a locality-based authentication mechanism. It ensures that SDP receives data from actual or expected BSN devices. As a result, it reduces the likelihood of receiving error packets that are depicted in Fig. 12.

**Figure 13:**
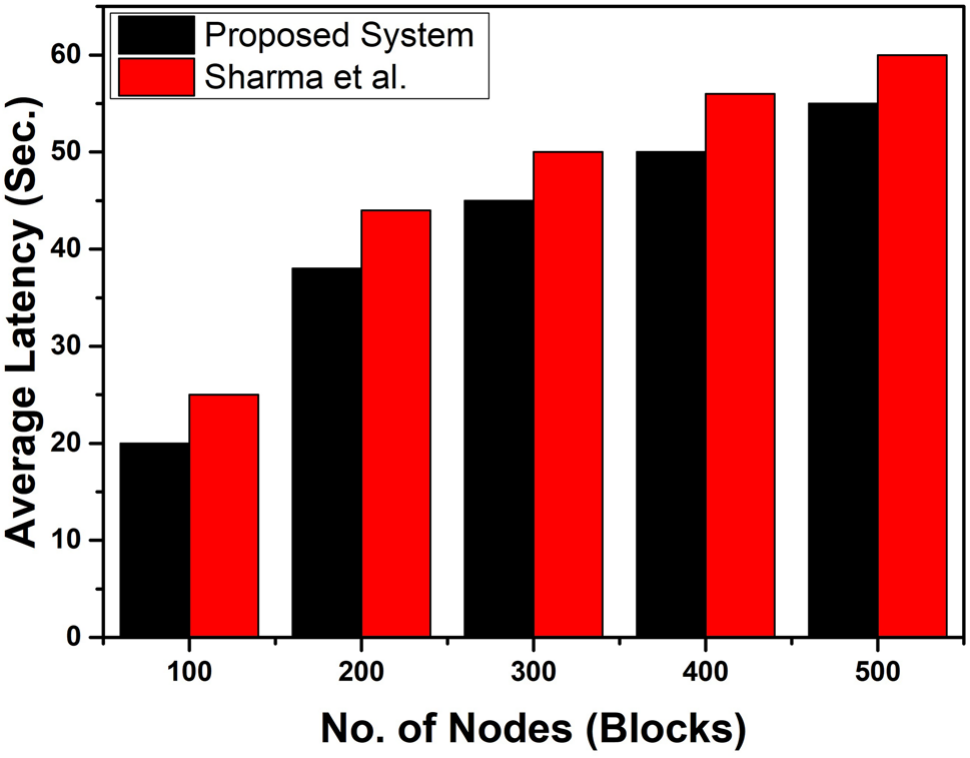
The Average Latency of the proposed architecture vs. IMTe-Healthcare^65^ with respect to the number of nodes.

On the other hand, Fig. 13 shows the average latency of our presented system. It compared with IMTe-Healthcare^65^ study with respect to the number of nodes. The average latency can be measured in time (sec.). Compared with each other, it is clear that our proposed method is capable of showing better performance for the transformation of data in the desired system. For analyzing the number of nodes (blocks), from low number to higher level number nodes, our presented system consistently shows better performance than the work^65^.

## Limitations, open challenges and future directions

Altering the traditional system is very rigid because it needs diligence and hard work. The lack of skilled and conscious people is an open challenge to continue such smart systems over the internet. Moreover, a security vulnerability is also a very ordinary event nowadays and occurs almost all the time. Agent-based healthcare system uses a computer or computer-like devices that are vulnerable or can be infected by many malicious viruses. These virus attacks can harm the system as this type of framework deals with confidential and delicate data. If these data are altered, it can collapse the total system–Ransomware, DDoS attack, Sybill attack, phishing. The study of these kinds of ideas requires a lot of expensive devices and precious time; as a result, researchers cannot perform the experimentation properly^66^.

In order to protect the health system monitoring mechanism, Blockchain can be a suitable solution to provide security to the data. As in this type of system, the main concern is to pass data securely and timely, the first and foremost concern is to protect the data from being manipulated by intruders. Further, Blockchain can also assist a system in detecting the possibility of attacks in a network which in turn facilitates data security. Again, machine learning makes a system secure by immense training and learning procedure The machine learns from data patterns that are being transferred. The healthcare systems can identify an abnormality in the data through this training process by leveraging the Multi-Factor Authentication process during the early stage of the system, which is the login phase. Through the use of Medical IoT along with ML, unauthorized access can be restricted from getting entry into the system. Thus continuous monitoring of the authorization can ensure the security of the system.

## Conclusion

This study demonstrates a patient-centric agent-based medical framework for IoMT networks, with the key focus being information security. SDN network BSN, Smartphone (Sensor Data Provider), Patient Centric Agent, Blockchain, and Healthcare Provider Interface make up the framework. We have analyzed different mechanisms for storing as well as accessing patients’ medical records considering both confidential/private and public records. In the proposed model, we use Hyperledger Fabric Network, which provides proper maintenance of private as well as public medical data. Secure communication of medical data has become a concerning issue when continuously observing a patient from a remote distance. This proposed architecture plays a great role in implementing this scenario. Here, we deployed a Hyper-ledger Fabric Network, and using this feature, the healthcare unit will be able to maintain its business policy easily in a unique way. According to the suggested architecture, we will develop a mobile application in the near future that can simply adapt this model and modify the smart contract process, allowing customers of IoMT networks to conveniently access services. This article can assemble the overall information such as patient’s private and public data, personal information, medicine-related information, and other necessary information related to patients, healthcare professionals, or healthcare providers to make a smart remote healthcare monitoring system.

## Data Availability

Data are available at https://github.com/anwarcse12028/SDNwPCA (On request). In addition, A. Rahman (anis_cse@niter.edu.bd) can be contacted for further data.
